# Detection of hepatitis E virus genotype 3 in an Algerian mouse (*Mus spretus*) in Portugal

**DOI:** 10.1007/s11259-024-10293-4

**Published:** 2024-01-20

**Authors:** Sérgio Santos-Silva, Danny Franciele da Silva Dias Moraes, Pedro López-López, Joana Paupério, João Queirós, António Rivero-Juarez, Laura Lux, Rainer G. Ulrich, Helena M.R. Gonçalves, Wim H.M. Van der Poel, Maria S.J. Nascimento, João R. Mesquita

**Affiliations:** 1 School of Medicine and Biomedical Sciences (ICBAS), University of Porto, Porto, Portugal; 2 State Department for the Environment of Mato Grosso (SEMA), Cuiabá, Brazil; 3 Unit of Infectious Diseases, Clinical Virology and Zoonoses, Instituto Maimonides de Investigación Biomédica de Córdoba (IMIBIC), Hospital Universitario Reina Sofia, Universidad de Córdoba (UCO), Cordoba, Spain; 4 Center for Biomedical Research Network (CIBER) in Infectious Diseases, Health Institute Carlos III, Madrid, Spain; 5 European Molecular Biology Laboratory, European Bioinformatics Institute, Welcome Genome Campus, Hinxton, CB10 1SD UK; 6 CIBIO—Centro de Investigação em Biodiversidade e Recursos Genéticos, InBIO Laboratório Associado, Universidade do Porto, Campus de Vairão, Vairão, 4485-661 Portugal; 7 BIOPOLIS Program in Genomics, Biodiversity and Land Planning, CIBIO, Campus de Vairão, Vairão, 4485-661 Portugal; 8 Departamento de Biologia, Faculdade de Ciências, Universidade do Porto, Rua Campo Alegre s/n, Porto, 4169-007 Portugal; 9 EBM, Estação Biológica de Mértola, Mértola, 7750-329 Portugal; 10 University of Greifswald, Domstraße 11, 17489 Greifswald, Germany; 11 Institute of Novel and Emerging Infectious Diseases, Friedrich-Loeffler-Institut (FLI), Federal Research Institute for Animal Health, Südufer 10, 17493 Greifswald-Insel Riems, Germany; 12 REQUIMTE, Instituto Superior de Engenharia do Porto, Porto, Portugal; 13 Biosensor NTech – Nanotechnology Services, Avenida da Liberdade, 249, 1º Andar, Lda, Lisboa, 1250-143 Portugal; 14 Quantitative Veterinary Epidemiology, Wageningen University, Wageningen, The Netherlands; 15 Department Virology & Molecular Biology, Wageningen Bioveterinary Research, Lelystad, The Netherlands; 16 Faculty of Pharmacy, University of Porto (FFUP), Porto, Portugal; 17 Epidemiology Research Unit (EPIUnit), Instituto de Saúde Pública da Universidade do Porto, Porto, Portugal; 18 Laboratory for Integrative and Translational Research in Population Health (ITR), Porto, Portugal

**Keywords:** HEV reservoir, One health, Rodent, Wildlife, Zoonosis

## Abstract

Virus monitoring in small mammals is central to the design of epidemiological control strategies for rodent-borne zoonotic viruses. Synanthropic small mammals are versatile and may be potential carriers of several microbial agents. In the present work, a total of 330 fecal samples of small mammals were collected at two sites in the North of Portugal and screened for zoonotic hepatitis E virus (HEV, species *Paslahepevirus balayani*). Synanthropic small mammal samples (*n* = 40) were collected in a city park of Porto and belonged to the species Algerian mouse (*Mus spretus*) (*n* = 26) and to the greater white-toothed shrew (*Crocidura russula*) (*n* = 14). Furthermore, additional samples were collected in the Northeast region of Portugal and included Algerian mouse (*n* = 48), greater white-toothed shrew (*n* = 47), wood mouse (*Apodemus sylvaticus*) (*n* = 43), southwestern water vole (*Arvicola sapidus*) (*n* = 52), Cabrera’s vole (*Microtus cabrerae*) (*n* = 49) and Lusitanian pine vole (*Microtus lusitanicus*) (*n* = 51). A nested RT-PCR targeting a part of open reading frame (ORF) 2 region of the HEV genome was used followed by sequencing and phylogenetic analysis. HEV RNA was detected in one fecal sample (0.3%; 95% confidence interval, CI: 0.01–1.68) from a synanthropic Algerian mouse that was genotyped as HEV-3, subgenotype 3e. This is the first study reporting the detection of HEV-3 in a synanthropic rodent, the Algerian mouse. The identified HEV isolate is probably the outcome of either a spill-over infection from domestic pigs or wild boars, or the result of passive viral transit through the intestinal tract. This finding reinforces the importance in the surveillance of novel potential hosts for HEV with a particular emphasis on synanthropic animals.

## Introduction

Hepatitis E virus (HEV) is a small nonenveloped single-stranded positive sense RNA virus that belongs to the *Hepeviridae* family, subfamily *Orthohepevirinae*, genus *Paslahepevirus.* species *balayani* (Purdy et al. 2022). In both developing and industrialized countries, HEV is the main cause of acute hepatitis, reported to infect around 20 million individuals and resulting in 3.3 million symptomatic cases and 44,000 fatalities annually worldwide (WHO 2022). Hepatitis E was identified as a zoonotic illness in the late 1990s and pigs were discovered to be the primary natural host of HEV genotypes 3 and 4 (Dalton et al. 2008). The intake of undercooked pork meat was soon linked to a significant number of autochthonous cases in several European countries (Pavio et al. 2015).

HEV (*Paslahepevirus balayani*) includes variants detected in humans, pigs, wild boar, deer, mongoose, rabbits, camels and other animals, with 8 genotypes recognized until today (HEV-1 - HEV-8), whereby HEV-3, HEV-4 and HEV-7 with zoonotic transmission (Smith et al. 2020). Genotypes HEV-3 and HEV-4 are transmitted via contact with infected animals, especially pigs, or mainly by consumption of contaminated pork meat products (Velavan et al. 2021). These two genotypes have been detected in *Sus scrofa domesticus* (domestic pigs) and *Sus scrofa scrofa* (wild boars), as well as in humans (Berto et al. 2012; de la Villalba et al. 2013; Santos-Silva et al. 2023). Despite numerous studies on HEV-3 and HEV-4, particularly in high-income countries where transmission of these two genotypes via the fecal-oral route and infections acquired through environmental contamination with animal feces is still mostly unknown, other potential transmission routes are being investigated, such as milk (King et al. 2018; Treagus et al. 2021; Santos-Silva et al. 2022).

In rats, the first description of HEV was in 1993 in the former Soviet Union, in a region that experienced a viral hepatitis outbreak in human in July-October 1989 having been pointed out to rodents a certain role in the process of the spread of the virus (Karetnyĭ et al. 1993). However, molecular detection and characterization of HEV RNA in Norway rats (*Rattus norvegicus*) only occurred in 2010 (Johne et al. 2010b). Moreover, currently a growing body of data shows evidence of the circulation of HEV-3 in small mammals, like rats and rabbits (Lack et al. 2012; Ryll et al. 2017).

Besides the initial reports of HEV-3 in small mammals, additional *Hepeviridae* members have also been found, namely *Rocahepevirus ratti*. Putative genotypes of rat Hepatitis E virus (species *Rocahepevirus ratti*; ratHEV) HEV-C3 and HEV-C4 were identified in *A. chevrieri* and *E. melanogaster*, respectively (Wang et al. 2018), however, there is a high likelihood of numerous additional orthohepeviruses being present in different rodent species and geographic locations (Wang et al. 2018). Genotypes of *Paslahepevirus balayani* species xhibit a greater divergence than other hepeviruses species (Reuter et al. 2020) and are distinctly different from genotypes HEV-C1 and HEV-C2 of *Rocahepevirus ratti* (Wang et al. 2020).

The zoonotic potential of rocahepeviruses is now recognized. Interestingly, ratHEV replication in a human-derived cell line was demonstrated (Jirintai et al. 2014; Li et al. 2015). Notwithstanding, experimental infection in monkeys and domestic pigs failed (Purcell et al. 2011; Cossaboom et al. 2012), however a recent study successfully inoculated ratHEV in *Rhesus* and *Cynomolgus* monkeys (Yang et al. 2022). Nevertheless, ratHEV infection has been linked to chronic hepatitis and acute hepatitis (Sridhar et al. 2018; Andonov et al. 2019). Additionally, ratHEV human infections have also recently been reported in Europe (Rivero-Juarez et al. 2022; Rodriguez et al. 2023), and IgG anti-ratHEV-reactive IgG antibodies have been detected in forestry workers in Germany and Japan (Dremsek et al. 2012; Li et al. 2013; de Cock et al. 2022).

Small mammals include animals of the order *Eulipotyphla* (formerly known as *Insectivora*) and the order *Rodentia*. They have extremely versatile habits, inhabiting various locations with the ability to settle between wild and urban environments (Bencatel et al. 2017). The full spectrum of small mammals, including synanthropic animals that have adapted to human environments, representing additional HEV reservoirs and playing a role in the epidemiology of zoonotic HEV is still far from being clarified. Synanthropic animals thrive in close proximity to human populations and may frequently come into contact with humans and their habitats, thereby increasing the potential for zoonotic transmission. Detecting spill-over infections in small mammals is valuable as it helps map the occurrence of HEV strains in different regions. Understanding the crucial role of synanthropic animals in the epidemiology of HEV is essential for developing effective prevention and control strategies, as they can serve as additional reservoirs and vectors for the virus. Consequently, comprehensive research that encompasses both wildlife and synanthropic animals is necessary to gain a complete understanding of the complex dynamics of HEV transmission.

In Portugal, HEV infection in humans has been widely reported (Berto et al. 2012; Mesquita et al. 2016; Moraes et al. 2022) and HEV RNA has been detected in domestic pigs and in wild mammals, such as wild boar and red deer (Moraes et al. 2022; Santos-Silva et al. 2023), however no studies have been ever carried out in small mammals. Hence the aim of this study was to perform a screening and to provide genetic characterization of HEV detected in synanthropic and wild small mammals from Portugal.

## Materials and methods

### Sampling and collection

**Fig. 1:**
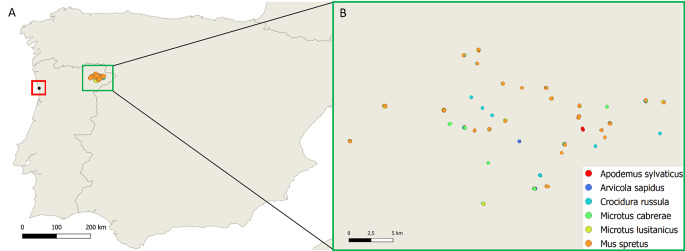
(**A**) Map of Portugal. Red and green squares highlight the sampling site of synanthropic and wild small mammals, respectively. (**B**) Location of the 24 sampling units centered in olive grove patches. Common names of small mammal species are as follows: wood mouse (*Apodemus sylvaticus*), southwestern water vole (*Arvicola sapidus*), greater white-toothed shrew (*Crocidura russula*), Cabrera’s vole (*Microtus cabrerae*), Lusitanian pine vole (*Microtus lusitanicus*) and Algerian mouse (*Mus spretus*)

A total of 330 fecal samples from synanthropic and wild small mammals were collected. Samples from synanthropic animals, represented by the wild species Algerian mouse (*Mus spretus*) (*n* = 26) and by the greater white-toothed shrew (*Crocidura russula*) (*n* = 14), were collected in the spring of 2014 from a public city park in Porto, Portugal (Fig. 1). The wild small mammal fecal samples (*n* = 290) were collected during ecological studies (Barão et al. 2022; Lux et al. 2023) from six different species in the spring of 2020 in the Northeast region of Portugal (Trás-os-Montes). Wild small mammal droppings were collected from 24 different sampling units, representing six different habitat types and were from one insectivore (greater white-toothed shrew, *Crocidura russula*, *n* = 47) and from five species of rodents, including two species of the family *Muridae* (wood mouse, *Apodemus sylvaticus*, *n* = 43; Algerian mouse, *Mus spretus*, *n* = 48) and three species of the family *Cricetidae* (southwestern water vole, *Arvicola sapidus*, *n* = 52; Cabrera’s vole, *Microtus cabrerae*, *n* = 49; Lusitanian pine vole, *Microtus lusitanicus*, *n* = 51).

Wild small mammal species of the samples were previously determined by molecular analysis of a short 12 S rRNA gene fragment to genetically identify the host species (Barão et al. 2022). Fresh samples were immediately transported to the laboratory and kept frozen at -20 °C until further analysis.

### Sample preparation and RNA extraction

Individual fecal suspensions (10% in phosphate-buffered saline, pH 7.2) and 0.2 g sterile silicone microbeads (Precellys Lysing kits, Bertin Technologies SAS, Montigny-le-Bretonneux, France) were prepared. Fecal samples were shredded with plastic pestles, vortexed for 5 min using the cell disruptor Disruption Genie (Scientific industries, Inc., Bohemia, NY, USA) and centrifugated at 8,000 × g for 5 min according to previously described methods (Mesquita et al. 2010). Nucleic acid extraction was carried out from 140 µL of clarified supernatants using the automatic nucleic acid extraction machine QIAcube® (Qiagen, Hilden, Germany) and QIAamp® viral RNA mini kit (Qiagen, Hilden, Germany). The RNA was eluted in 50 µL of RNase-free ultrapure water and kept at -80 °C until further use.

### Amplification of HEV RNA

All RNA extracts were tested for HEV RNA by a pangenotypic nested RT-PCR assay that targets the open reading frame (ORF) 2 region that encodes the viral capsid protein of the HEV genome (exclusively for HEV genotypes of species *Paslahepevirus balayani*, amplifying a 467 nucleotides (nt)-long genome fragment in the second round spanning between nt 5930–6334 (Frías et al. 2021).

RNA extracts were also tested by a broad-spectrum nested RT-PCR assay targeting the RNA-dependent RNA-polymerase (*RdRp*) gene of the ORF1 region of the HEV genome (nt 331–334 ) spanning nt 4285–4616 (numbering according to genotype 3 strain Meng accession number AF082843), that was developed for detection of novel hepeviruses (Johne et al. 2010b). For the first round, Qiagen One-Step™ RT-PCR kit (Qiagen®, Hilden, Germany) was used for both assays and for the second round, 5 µL of the first-round products were used as templates with GoTaq® (Promega™, WI, U.S.A.), all according to the manufacturer’s instructions. The WHO PEI 6329/10 subgenotype 3a standard (accession number AB630970, provided by the Paul Ehrlich-Institute, Langen, Germany) was used as a positive control and RNase-free water as negative control. Amplification reactions, with the corresponding positive and negative controls (nuclease – free water), were conducted in Bio-Rad T100TM Thermal Cycler. The conditions for first rounds were an initial reverse transcription (RT) step for 15 min at 45ºC followed by 3 min at 95ºC (enzyme activation, denaturation of template DNA). For the pangenotypic nested RT-PCR assay the thermal profile includes 40 cycles of 95 ºC for 15 s, 52 ºC for 15 s, and 72 ºC for 2 s, with a final elongation at 72 ºC for 10 min. For the broad-spectrum nested RT-PCR assay the same conditions were followed except the annealing temperature was set as 50 ºC. Both second rounds followed the same conditions, excluding the RT step.

If samples were positive for any of the above molecular approaches, HEV RNA quantification was also attempted using a broad-spectrum real-time RT-PCR (RT-qPCR) assay targeting the open reading frame ORF3 region with primers/probe (TaqMan) as previously described (Jothikumar et al. 2006). The RT-qPCR was performed using iTaq Universal Probes One-Step Kit (Bio-Rad Laboratories, USA) at a final total of 20 µL reaction mixture volume in a CFX Connect Real-Time thermocycling System (Bio-Rad Laboratories, USA). The thermal cycling regimen for the RT-qPCR reaction included initial reverse transcription (RT) at 50 °C for 10 min, followed by a simultaneous step for reverse transcriptase inactivation and the initial denaturation of cDNA at 95 °C for 3 min. Subsequently, 45 cycles of amplification were carried out, involving denaturation at 95 °C for 15 s and annealing/extension at 55 °C for 15 s.

### Sequencing and phylogenetic analysis

RT-PCR products were separated by electrophoresis at 100 V for 40 min on a 1.5 % agarose gel stained with Xpert Green Safe DNA gel dye (GriSP®) and documented using the ChemiDoc XRS system with ImageLab software (Bio-Rad, Hercules, CA, USA). Bands of the expected size were excised and treated enzymatically to remove unincorporated primers and nt using Illustra™ ExoProStar™ (Sigma Aldrich® Darmstadt, Germany). Amplicons were further sequenced in both directions with the dideoxy-chain termination method using the BigDye Terminator v1.1 Cycle Sequencing kit (PE Applied Biosystems, Foster City, CA, USA). Sequence editing and multiple alignments were performed with the BioEdit software package, version 2.1 (Ibis Biosciences, Carlsbad, CA, USA). Aligned sequences were compared to sequences found in the NCBI (GenBank) nucleotide database, retrieved on 1 February 2023 (http://blast.ncbi.nlm.nih.gov/Blast). Phylogenetic analysis was performed using MEGA version X software (Kumar et al. 2018). The maximum-likelihood (ML) approach was used to infer this analysis (Tamura 1992; Kumar et al. 2018), and Tamura-Nei model was used to estimate the maximum likelihood (ML) bootstrap values using 1000 replicates. The Tamura-Nei model was determined by MEGA version X (Kumar et al. 2018) as the best replacement. Further typing was performed with the HEVnet genotyping tool (Mulder et al. 2019) to identify genotypes/subgenotypes of HEV.

## Results

**Fig. 2:**
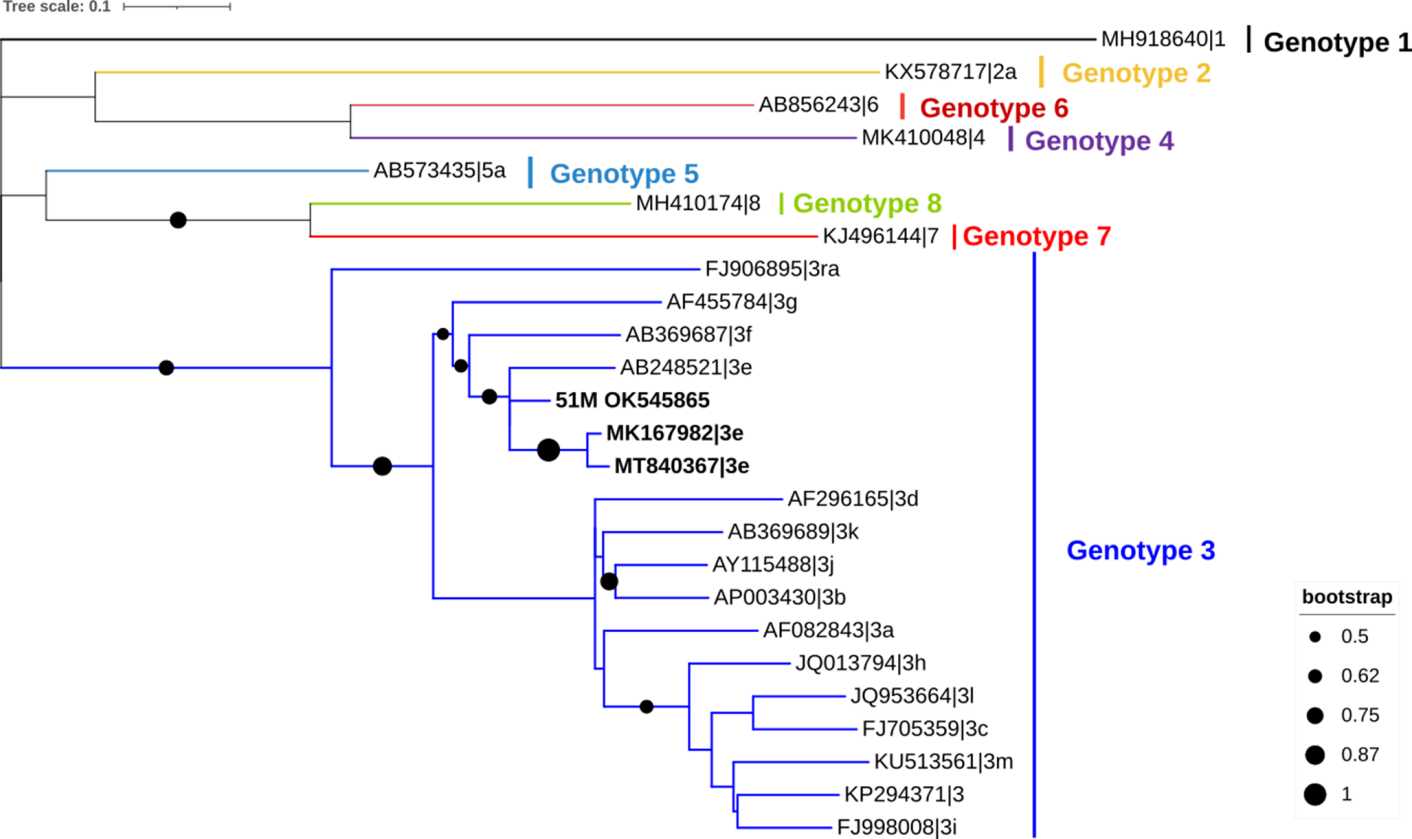
Phylogenetic analysis of HEV sequence found in a synanthropic Algerian mouse from Portugal. HEV-3 found in this study (OK545865) and the closest related variants of human (MK167982, from United Kingdom) and animal (MT840367, wild boar from Italy) origin and their respective accession numbers are highlighted in bold in the tree inferred using the MEGA X software and the Interactive Tree of Life (iTOL) based on 50 nucleotide HEV sequences as well as 49 strains of various genotypes obtained from GenBank.

From the 330 fecal samples tested, one was positive for HEV RNA by the pangenotypic nested RT-PCR assay that targets the ORF2 (0.3%; 95% confidence interval, CI: 0.01–1.68) (Table 1). No sample was positive using the broad-spectrum nested ORF1-targeting RT-PCR assay and the in-house broad-spectrum RT-qPCR assay targeting the ORF3 region. The HEV RNA positive stool sample originates from a synanthropic Algerian mouse, which represented an occurrence of 1.4% (95% CI: 0.03–7.3) for this species when considering both synanthropic and wild Algerian mouse and 2.5% (95% CI: 0.06–13.16) for the total of the synanthropic samples collected. The respective generated consensus sequence was identified as HEV subgenotype 3e by Blast search. Phylogenetic analysis based on the 467 nt-long partial region of the ORF2 confirmed clustering with HEV-3 subgenotype e (Fig. 2), being closely related to variants of human (MK167982, from United Kingdom) and animal (MT840367, wild boar from Italy) origin. Furthermore, pairwise nt sequence similarity of the positive sample and the positive control was 81.2%. The sequence of HEV detected in this study is available in GenBank database under accession number OK545865. The mean pairwise distances analysis between each reference HEV sequences, as recommended for HEV subtyping studies (Smith et al. 2020), and the positive (51 M) sample is described in Table 2. No sample was positive for ratHEV RNA.

**Table 1:** HEV RNA* detection rate in the different small mammal species studied and the results of HEV genotype characterization

Small mammals		Results			
		No. of wild small mammals’ positive samples/No- of analysed samples (detection rate in %, 95% CI)	HEV	No. of synanthropic small mammals’ positive samples/No- of analysed samples (detection rate in %, 95% CI)	HEV
Family	Species (common names)	HEV (ORF1 RT-PCR)	Genotype/subgenotype	HEV (ORF2 RT-PCR)	Genotype/subgenotype
Muridae	*Apodemus sylvaticus* **(wood mouse)**	0/43	ND	0/0	ND
	*Mus spretus* **(Algerian mouse)**	0/48	ND	1/26 (3.9%, 0.1–19.6)	3e
Total		0/91	ND	1/26 (3.9%, 0.1–19.6)	3e
Cricetidae	*Arvicola sapidus* **(southwestern water vole)**	0/52	ND	0/0	ND
	*Microtus cabrea* **(Cabrera’s vole)**	0/49	ND	0/0	ND
	*Microtus lusitanicus* **(Lusitanian pine vole)**	0/51	ND	0/0	ND
Total		0/152	ND	0/0	ND
Soricidae	*Crocidura russula* **(greater white-toothed-shrew)**	0/47	ND	0/14	ND
Total		0/290	ND	1/40 (2.5%, 0.06–13.16)	3e

ORF – Open reading frame; ND – Not determined; CI – Confidence Interval; HEV – hepatitis E virus

*based on the detection of HEV RNA by nested RT-PCR targeting ORF2

**Table 2:** The analysis involved 50 nucleotide sequences and focused on the number of base substitutions per site between sequences, as well as the mean pairwise distances between each HEV genotype/sugenotype and between 51 M. The final dataset contained 428 positions in total

	Pair Wise Distance (%)		Pair Wise Distance (%)
Accession number|HEV genotypes/subgenotypes	51 M	Accession number|HEV genotypes/subgenotypes	51 M
FJ457024|1	78.27	FJ998008|3i	84.35
MH918640|1	77.10	AY115488|3j	85.28
M73218|1a	79.44	AB369689|3k	83.88
L08816|1b	78.04	JQ953664|3 L	82.94
X98292|1c	78.97	KU513561|3 m	83.88
AY230202|1d	78.74	FJ906895|3ra	81.31
AY204877|1e	78.04	AB369688|4	78.97
JF443721|1f	78.27	MK410048|4	79.67
LC225387|1 g	80.14	AB197673|4a	79.44
KX578717|2a	77.10	DQ279091|4b	77.80
MH809516|2b	79.67	AB074915|4c	78.74
AB290313|3	88.08	AJ272108|4d	77.57
LC260517|3	84.11	AY723745|4e	79.21
MF959764|3	83.64	AB220974|4f	76.17
MF959765|3	83.64	AB108537|4 g	77.34
MK390971|3	81.78	GU119961|4 h	78.27
KP294371|3	83.88	AB369690|4i	79.21
AF082843|3a	83.41	AB573435|5a	80.56
AP003430|3b	85.75	AB856243|6	77.34
FJ705359|3c	82.94	AB602441|6a	77.80
AF296165|3d	82.89	KJ496144|7	81.07
AB248521|3e	90.65	KJ496143|7a	78.27
AB369687|3f	89.25	MH410174|8	75.93
AF455784|3 g	85.51	KX387865|8a	76.64
JQ013794|3 h	82.48		

## Discussion

The potential role and impact of small mammals as a HEV reservoir is still largely unknown. The present study offers the first molecular-based proof of the presence and identification of the zoonotic HEV-3 in a synanthropic Algerian mouse in Portugal.

The first molecular identification of a hepevirus (ratHEV, species *Rocahepevirus ratti*) in rodents was in Norway rat feces and liver samples in Germany in 2010 (Johne et al. 2010a, b). Since then ratHEV strains have been detected in rats across at least three continents (Asia, America, and Europe), indicating that rats are infected with the virus throughout a large geographic range (Reuter et al. 2020). In addition, ratHEV has been found in a variety of wild rat species, including Norway rats (*Rattus norvegicus*) and Black rats (*Rattus rattus*) in many European countries (Johne et al. 2010a; Ryll et al. 2017; Simanavicius et al. 2018; Murphy et al. 2019), United States of America (Purcell et al. 2011), Hong Kong (Sridhar et al. 2021), Indonesia (Mulyanto et al. 2014; Primadharsini et al. 2018). Furthermore, ratHEV has also been detected in synanthropic rats (Porea et al. 2023), and in humans (Rivero-Juarez et al. 2022) from Europe. In the present study, ratHEV was not detected in any of the 290 wild small mammal fecal samples that included one insectivore and five rodent species. Further studies assessing urban rats/rodents (*Rattus* sp.) should be conducted in Portugal in order to estimate the zoonotic risk of infection, considering the findings in neighboring countries, such as France and Spain.

Nevertheless, in this study, HEV-3 subgenotype 3e was detected in the fecal sample of a synanthropic Algerian mouse, that goes in accordance to results from previous studies that also report this human pathogenic genotype in small mammals (Lack et al. 2012). However, no accurate assessment of the variety of hepevirus species/genotypes circulating in wild small mammals in Portugal can be made due to the extremely low number of positive samples. Furthermore, the positive sample was identified using the pangenotypic nested RT-PCR assay that targets the ORF2 region that encodes the viral capsid protein of the HEV genome and specific to the species *Paslahepevirus balayani* and not with the broad-spectrum nested RT-PCR assay targeting the *RdRp* gene of the ORF1 region of the HEV genome and the broad-spectrum RT-qPCR assay targeting the ORF3 region. These differences may be attributed to the genetic variability of the virus, which may have hindered detection with the other sets of primers.

According to the HEVTool (Mulder et al. 2019) and phylogenetic analysis, the HEV strain detected in the Algerian mouse was associated with strains that cause more severe illness in humans (Subissi et al. 2019). Since synanthropic small mammals live in close association with people and benefit from their surroundings and activities and although the uncertainty in how HEV behaves in synanthropic and wild small mammals, there is some concern that it could spread and infect humans and other species. However, as previously mentioned, the HEV positive sample found in this study could potentially be result of a spill-over infection or of passive viral transit through the intestinal tract, which is highly unlikely to be transmitted to humans or animals. In a study from Italy, rats inhabiting a pig farm were found to have HEV-3e (the same subgenotype found in pigs), highlighting the possible involvement of rats in the spread of this virus (De Sabato et al. 2020) or at least a spill-over effect that enables the tracking of this particular virus strain within a specific geographic area. Interestingly, in previous studies, HEV-3 RNA was detected in intestinal contents, but not in the liver of mice from a pig farm house and was considered to be a virus that had entered the intestine from ingested feces of pigs rather than infection of mice (Grierson et al. 2019). In addition, studies show the absence of genotype HEV-3 in liver tissue samples from wild rats, supporting the argument that rats, like mice, are only accidental hosts of HEV (Takahashi et al. 2022). Nevertheless, further investigations may be required before discarding the possibility of HEV replication in rats. A study reported wild-type and immunodeficient mice being resistant to HEV-3 infection, yet HEV RNA and anti-HEV antibodies were detected in rats inoculated with HEV, indicating a successful infection (Schlosser et al. 2019).

In our study a low HEV detection rate was seen in synanthropic and wild small mammals (0.3%), with HEV-3 being identified. A similar detection rate (0.2%) has been reported in a study that detected HEV-3 in one Norway rat in a survey conducted in 11 European countries (1/508, from Belgium) (Ryll et al. 2017), while in USA 7.85% (35/446) of the *Rattus* spp. surveyed positive for HEV-3 having some been classified as subgenotype 3a (Lack et al. 2012). Besides rats, HEV-3a has also been detected in another small mammal (namely yellow-necked field mouse, *Apodemus flavicollis*) in Europe (Prpić et al. 2019). Furthermore, this HEV genotype has been identified in other mammal species, such as wild boar, from the same region of Portugal as our study (Mesquita et al. 2016; Santos-Silva et al. 2023). Therefore, the question whether rodents are truly natural reservoirs of human HEV or act as only intermediate hosts or bioindicators is not yet conclusively answered (Kenney 2019; Wang et al. 2020).

While the detection rate in this study is relatively low, the results suggest a potential susceptibility of these small rodents to HEV infection. Furthermore, strikingly HEV was only detected in a synanthropic rodent that is closely associated with people and that benefits from people surroundings and activities, whereas no non-synanthropic wild small mammal have shown evidence of HEV RNA. As such, the present study on HEV in small mammals of Portugal as a potential source for hepevirus transmission highlights the necessity for further research to improve risk assessment for human health.

In conclusion, this is the first time that HEV subgenotype 3e was identified in an Algerian mouse. However, the HEV isolate identified is most likely the result of a spillover infection from domestic pigs, or possibly the result of passive viral transit through the intestinal tract. Moreover, our study is the first hepevirus report in Portuguese small mammals, raising questions about their susceptibility to HEV and their role in HEV epidemiology.

## Data Availability

The data that support the findings of this study are available from the corresponding author upon reasonable request.
